# First Trimester Placental Biomarkers for Pregnancy Outcomes

**DOI:** 10.3390/ijms25116136

**Published:** 2024-06-02

**Authors:** Martina Cristodoro, Martina Messa, Giovanni Tossetta, Daniela Marzioni, Marinella Dell’Avanzo, Annalisa Inversetti, Nicoletta Di Simone

**Affiliations:** 1 Department of Biomedical Sciences, Humanitas University, Via Rita Levi Montalcini 4, Pieve Emanuele, 20072 Milano, Italy; martina.cristodoro@humanitas.it (M.C.);; 2 Department of Experimental and Clinical Medicine, Università Politecnica delle Marche, 60126 Ancona, Italy; 3 Humanitas San Pio X, Via Francesco Nava 31, 20159 Milan, Italy; 4 IRCCS Humanitas Research Hospital, Via Manzoni 56, Rozzano, 20089 Milan, Italy

**Keywords:** preeclampsia, gestational diabetes mellitus, intrauterine growth restriction, pregnancy outcomes

## Abstract

The placenta plays a key role in several adverse obstetrical outcomes, such as preeclampsia, intrauterine growth restriction and gestational diabetes mellitus. The early identification of at-risk pregnancies could significantly improve the management, therapy and prognosis of these pregnancies, especially if these at-risk pregnancies are identified in the first trimester. The aim of this review was to summarize the possible biomarkers that can be used to diagnose early placental dysfunction and, consequently, at-risk pregnancies. We divided the biomarkers into proteins and non-proteins. Among the protein biomarkers, some are already used in clinical practice, such as the sFLT1/PLGF ratio or PAPP-A; others are not yet validated, such as HTRA1, Gal-3 and CD93. In the literature, many studies analyzed the role of several protein biomarkers, but their results are contrasting. On the other hand, some non-protein biomarkers, such as miR-125b, miR-518b and miR-628-3p, seem to be linked to an increased risk of complicated pregnancy. Thus, a first trimester heterogeneous biomarkers panel containing protein and non-protein biomarkers may be more appropriate to identify and discriminate several complications that can affect pregnancies.

## 1. Introduction

The placenta is a temporary organ that forms in the uterus during pregnancy. It facilitates the exchange between the mother and fetus. Throughout pregnancy, the placenta performs numerous functions, including adapting the mother’s physiology, ensuring immunological acceptance, and providing nourishment and support to the developing embryo [1]. The placental villi, immersed in maternal blood, act as transport units, delivering nutrients and oxygen to the fetus while removing waste products [2]. Over the nine months of gestation, these villi undergo significant morphological changes. Initially, mesenchymal villi develop into highly vascularized structures to efficiently extract substances from the maternal circulation [3]. Additionally, the placenta secretes hormones into the maternal bloodstream, profoundly altering the maternal metabolism to support pregnancy, mobilize nutrients, facilitate childbirth and prepare for lactation. Some of these hormones also enter the fetal circulation, influencing the fetal development, growth and delivery timing [4].

The placental function and role in complicated pregnancies are still poorly understood. Failures in placental formation can hinder embryonic growth and development, contributing to complications, such as miscarriage, stillbirth, pre-term labor, intrauterine growth restriction (IUGR) and preeclampsia [5,6]. These disorders, which are often caused by a combination of fetal abnormalities and maternal factors, frequently involve placental defects and inadequate adaptation and remodeling of the uterine vascular bed, leading to malperfusion, particularly in severe IUGR and early-onset preeclampsia [7,8]. For instance, after analyzing the placenta of women affected by gestational diabetes mellitus (GDM), it was found that hyperglycemia altered the placental morphology and physiology [9]. In detail, increased placental weight and volume, villous edema, higher vasculogenesis rate and hypoperfusion are indicative of placental modifications in GDM [10,11]. Endothelial dysfunction, oxidative stress, altered cytotrophoblast invasion and decidualization may contribute to preeclampsia (PE) and intrauterine growth restriction (IUGR) development, determining placental dysfunction [12,13,14]. Thus, most adverse pregnancy outcomes, which become symptomatic in the third trimester, can be linked with earlier placental dysfunction, which generally develops during the first trimester [15].

Placental abnormalities not only increase the morbidity and mortality for both mother and fetus but can also negatively impact long-term health. For example, mothers with preeclampsia and infants with growth restriction face higher risks of developing type 2 diabetes, hypertension or cardiovascular disease later in life [2].

In this scenario, studying the placenta becomes essential not only for understanding the etiology of some adverse pregnancy outcomes but also for the identification of at-risk pregnancies to improve their management, therapy and prognosis [16,17].

Nowadays, the diagnosis of placental dysfunction is still based on nonspecific clinical, ultrasound and laboratory findings [18]. The aim of this review was to determine the possible placental biomarkers, which can be used to identify at-risk pregnancies in the first trimester.

The required characteristics of a good biomarker are a specific correlation with the disease; adequate predictability regarding the type of treatment and the response; the ability to be measured accurately and quickly; and being relatively insensitive to sampling errors [19].

## 2. Protein Placental Biomarkers

In this section, we summarize the studies reported in the literature regarding protein placental biomarkers (summarized in Table 1).

### 2.1. sFLT1 (Soluble Fms-Like Tyrosine Kinase 1), PLGF (Placental Growth Factor) and sFLT1/PLGF Ratio

PLGF and sFLT1 have essential roles in placentation since they can regulate angiogenesis [20]. In fact, PLGF is a proangiogenic factor, while sFLT1 is an antiangiogenic factor [20]. In detail, sFLT1 is a non-membrane soluble receptor that is able to link the Vascular Endothelial Growth Factor (VEGF) and also PLGF, resulting in reduced angiogenesis. Abnormal placentation is linked to higher levels of sFLT1 and reduced levels of PLGF. The increased production of sFLT1 promotes vasoconstriction and raises maternal blood pressure. These mechanisms are activated in a hypoxic condition [21]. In contrast, the reduced proangiogenic factor (PLGF) levels determine vasodilatation and contribute to decreased maternal blood pressure [21]. Recently, it was demonstrated that lower levels of PLGF may also be explained by its binding to sFLT1. Thus, high levels of sFLT1 determine lower levels of serum PLGF [22].

Several studies demonstrated that altered levels of these two proteins are associated with increased risks of PE, IUGR and preterm birth [23,24,25]. In physiologic pregnancies, sFLT1 levels increase as the gestation proceeds, while in women affected by pregnancy complications, sFLT1 levels increase earlier [21]. Similarly, PLGF levels decrease during pregnancy. However, in women affected by PE, PLGF levels decrease prematurely [26]. Nowadays, PLGF is already used in the PE prediction algorithm in the first trimester according to the Fetal Medicine Foundation algorithm [27]. Differently from PLGF, sFLT1 is not a valid tool in the prediction of PE in the first trimester because it starts to increase in the second trimester [28]. In the second and third trimesters, the sFTL1/PLGF ratio is an important tool in the prediction of PE, with a higher specificity than PLGF alone [29]. Nowadays, different guidelines recommend the use of this ratio in the prediction of PE [30]. In particular, in the first trimester, PLGF less than 12 pg/mL is associated with severe placental dysfunction and, consequently, a higher risk of PE. In contrast, PLGF higher than 100 pg/mL is correlated with the absence of placental dysfuction [30]. In the second and third trimesters, the sFLT1/PLGF ratio is fundamental in PE prediction. In detail, if the ratio is lower than 38, the risk of developing PE within one week is low. In contrast, if the ratio is higher than 38, the risk of developing PE within four weeks is high [30]. Several studies demonstrated that in the second and third trimesters, the sFLT1/PLGF ratio was also associated with an increased risk of preterm labor and IUGR [31].

### 2.2. PAPP-A (Pregnancy-Associated Plasma Protein-A)

PAPP-A is an important marker in screening tests for aneuploidy as β-hCG (β human chorionic gonadotropin) [32]. It was demonstrated that PAPP-A has a key role in placental growth and trophoblast invasion. In particular, PAPP-A cleaves IGFBP4 (insulin growth factor binding protein 4), releasing bioactive IGF and promoting growth [33]. PAPP-A levels increase during pregnancy, with higher concentration at term and a rapid reduction after delivery [34]. Several studies demonstrated that lower levels of PAPP-A detected during the first trimester screening were associated with a higher risk of complicated pregnancy (PE, IUGR, preterm birth) [35,36]. For instance, a PAPP-A of less than 0.4 multiple of the median (MoM) is predictive of third-trimester complications, such as preeclampsia, IUGR or preterm birth [37].

Based on these studies, PAPP-A seems to be a valid marker in predicting adverse pregnancy outcomes. Recently, a study confirmed these results, demonstrating that low PAPP-A levels in the first trimester of pregnancy are associated with a higher risk of having poor pregnancy outcomes, such as a preterm delivery and PE [36]. In contrast, elevated PAPP-A levels in the first trimester of pregnancy have been associated with a higher risk of developing placenta accreta spectrum disorders (PASs). In particular, a median PAPP-A of 1.96 MoM in women with placenta previa with PAS was significantly higher than a median PAPP-A of 0.89 MoM in women with placenta previa without PAS [38]. Despite that, the evaluation of PAPP-A alone in the first trimester cannot discriminate preterm delivery and PE occurrence; thus, this marker should be used in combination with other markers. These results confirmed a previous study conducted in 2019 by Xu et al. [39]. In this scenario, it appears that PAPP-A may be used also as an indicator for disease monitoring.

### 2.3. β-hCG (Human Chorionic Gonadotropin)

β-hCG plays a key role during feto-placental development in inducing angiogenesis, promoting cytotrophoblast cells fusion and syncytiotrophoblast differentiation, and reducing the immune system activity to promote fetal growth [40]. Research showed that villous angiogenesis in early pregnancy is regulated by β-hCG. This hormone plays a significant role in vascular adaptation during embryo implantation and early placenta formation [41]. Berndt et al. demonstrated that hCG promotes the migration of uterine endothelial cells and the formation of capillary structures in a 3D angiogenesis model, thus stimulating angiogenesis [42]. Additionally, Reisinger et al. found that hCG influences angiogenesis-related factors, such as vascular endothelial growth factor (VEGF) [43].

In this scenario, the importance of this protein in pregnancy and in maternal and fetal outcomes is clear. In 2023, when analyzing a population of Turkish women, it was found that high levels of β-hCG in the first trimester were associated with a higher risk of GDM, PE and preterm delivery [40]. Moreover, Younesi et al. also confirmed that high levels of β-hCG were associated with an increased risk of GDM [44]. In particular, levels of free βHCG MoM  > 5 are linked to a higher risk of GDM; in contrast, levels of free βHCG MoM < 0.2 are associated with a higher risk of pregnancy-induced hypertension [44]. This result is in contrast with other studies reporting that low levels of β-hCG are associated [45] or not associated [46] with GDM. In 2021 a meta-analysis conducted by Zhang and his group demonstrated that β-hCG had a predictive function for PE in the early second trimester, but not in the first trimester [47]. Thus, these contrasting data require other studies.

### 2.4. PP13 (Placental Protein 13)

PP 13 is secreted by syncytiotrophoblast and is involved in vascularization and placental implantation. Its levels increase slowly during pregnancy [48]. It was shown to induce the apoptosis of activated T cells in vitro and to target and kill T cells and macrophages in the maternal decidua in situ, indicating significant immune functions [49].

In the literature, several studies demonstrated that PP13 levels were lower in women who developed PE than women who did not develop PE [50]. This was also demonstrated by a recent meta-analysis conducted on 14 studies that aimed to evaluate the role of PP13 in predicting PE in the first trimester [51].

PP13 seems to also have a predictive role for GDM. In 2020, a group of researchers analyzed a population of 205 women (185 controls and 20 women who developed GDM). They found that PP13 levels were significantly lower in the first trimester in women who developed GDM than in controls [52]. Specifically, in women who developed GDM, the PP13 levels were 229 pg/mL (180–335) vs. 414 pg/mL (385–451) in controls [52].

This study underlined that the prediction of GDM by PP13 alone was poor and it was improved when combined with other biomarkers.

### 2.5. ADAM-12 (A Disintegrin and Metalloproteinase-12)

ADAM-12 has an important role in trophoblast migration and invasion [53]. In 2022, Ratnik and his group analyzed the serum samples of 53 patients (22 developed PE, 31 were controls) in order to find the predictive value of ADAM-12. They found that ADAM-12 levels were higher in both the first and second trimesters in women who developed PE later than in controls. In particular, in the first trimester, the ADAM-12 concentrations were 79.1 ng/mL [69.1–98.5] in controls vs. 110.8 ng/mL [97.8–202.3] in women who developed PE [53]. This significant difference was not found in the third trimester [54]. This study seems not to confirm the results of Yu et al.’s study in 2017, who demonstrated that a lower concentration of ADAM-12 was linked to an increased risk of developing PE [55].

Moreover, few studies evaluated the possible predictive role of this protein in IUGR. In detail, in pregnancies complicated by IUGR, the ADAM-12 levels were significantly lower than in women who did not develop IUGR [56,57]. Particularly, Cowans et al. demonstrated a linear relationship between ADAM-12 levels and the severity of IUGR. Furthermore, they also found significantly higher levels of ADAM-12 in pregnancies complicated by a predicted large for gestational age (LGA) infant [57]. Recently, Andres et al. evaluated ADAM-12 levels at 36 weeks. They demonstrated that women who had small for gestational age (SGA) infants had significantly reduced ADAM-12 levels compared with controls, but they did not find any difference between women who had PE and controls [58]. In contrast, in other studies, it was demonstrated that ADAM-12 levels in the first trimester were not associated with an increased risk of IUGR [59,60].

### 2.6. SHBG (Sex Hormone Binding Globulin)

SHBG is a glycoprotein that binds sex steroids. By binding testosterone, SHBG serves as a sensitive indicator of androgen levels in women [61]. Research demonstrated that SHBG levels are closely associated with central obesity, BMI, HDL, apolipoprotein B (apoB) and insulin levels in women, with the strongest correlation being with insulin resistance [61]. A decrease in SHBG levels was identified as an independent risk factor for insulin resistance in women with GDM [62]. Its levels are altered in different conditions, such as thyroid disorders, prostate or breast cancer, liver disorders and hypo/hyperandrogenism [63]. Several studies demonstrated that SHBG levels were decreased in patients affected by insulin resistance and T2DM (type 2 diabetes mellitus) [64,65].

In 2022, analyzing 229 patients in the sub-Saharan region, Basil et al. evaluated SHBG levels in the first trimester in 180 controls and 49 women who developed late GDM. They found a significantly lower concentration of SHBG in the case women (104.7 ± 61.6 nmol/L) than in controls (265.2 ± 141.5 nmol/L) [66]. These results were also confirmed by Zhang et al. in 2018 in China on 443 patients [67]. In contrast, some studies demonstrated that there was no association between SHBG levels and GDM. In detail, in 2019, Correa et al. analyzed SHBG levels in the first trimester in 80 controls and 16 women who developed GDM and found no difference between these two groups [68]. In the literature, the results are contrasting [69,70,71].

### 2.7. Afamin

Afamin is a vitamin-E-binding glycoprotein [72]. Vitamin E is an important antioxidant [73]. Higher levels of oxidative stress lead to increased levels of afamin; higher concentrations of this protein are found in endometriosis, metabolic syndrome, insulin resistance, PCOS (polycystic ovary syndrome) and T2DM [74]. Considering the correlation between afamin levels and inflammation, different studies analyzed the role of afamin in predicting pregnancy complications.

In 2018, for the first time, the association between afamin concentration and GDM was studied in 59 women who developed GDM and 51 controls. In detail, women who developed GDM had higher levels of afamin in the first trimester compared with controls. Analogously, also in the second trimester, afamin levels were significantly higher in women who developed GDM compared with controls [75]. Afamin also seems to have a predictive role in PE. In 2018, Tramontana et al. evaluated the afamin concentration in the first trimester in 474 women: 84 women who developed gestational hypertension, 30 women who developed PE, 107 women who developed IUGR, 44 women who developed preterm birth and 209 women who developed GDM. It was found that the median afamin concentration was significantly higher in women who developed PE compared with controls (76 mg/L vs. 65 mg/L). Moreover, it was confirmed that the afamin concentration was higher in women who developed GDM compared with controls (80 mg/L vs. 69 mg/L) [76].

In gestational hypertension, IUGR and preterm birth, there were no significant differences in the afamin levels compared with controls [77]. The potential role of afamin as a predictive biomarker for PE in the first trimester was also confirmed by Gülücü et al., who analyzed a larger cohort of patients (118 patients: 74 who developed PE and 44 controls) [77]. Furthermore, afamin levels are higher in women who develop late PE in the second trimester and third trimester [77,78].

The role of afamin in predicting PE and GDM was summarized in a recent meta-analysis that when evaluating 11 studies, found that in the first trimester, afamin levels were significantly higher in both women who developed PE and in women who developed GDM. In the second and third trimesters, afamin levels were significantly higher in women affected by PE. In contrast, afamin levels seemed not to be associated with GDM in the second and third trimesters [79].

### 2.8. FABP4 (Fatty Acid Binding Protein 4)

FABP4 is a protein expressed by adipocytes, macrophages and placental endothelial cells [80]. In immune cells, FABP4 expression is stimulated by inflammation [81]. Moreover, FABP4 is a crucial component of the insulin counter-regulatory hormonal network that rises in response to hypoglycemia and promotes glucose production [82]. Several studies demonstrated the association between higher FABP4 levels and some diseases, such as T2DM, cancer and cardiovascular diseases [83].

Recently, Paiboonborirak et al. determined the role of FABP4 in predicting PE in the first trimester in combination with a Doppler of uterine artery PE. Among 330 patients, 21 developed PE. It was found that women who developed late PE had significantly higher levels of FABP4 compared with controls [84]. The correlation between FABP4 and PE was also found in a population study of 22 women who developed PE and 72 controls. This study demonstrated that FABP4 levels were higher in women who developed PE, both in the first trimester and in the second trimester [85].

Furthermore, several studies also analyzed the possible predictive role of FABP4 in GDM. In 2021, a nested case–control study conducted in China on 135 women who developed GDM and 135 controls found that FABP4 levels were significantly higher in women who developed GDM compared with controls, both in the first trimester [53.3 (33.1~93.2) ng/L vs. 42.4 (32.6~63.8) ng/L] and in the second trimester [53.8 (36.8~94.1) ng/L vs. 41.6 (33.4~64.1) ng/L] [86]. The role of this protein in predicting GDM in the first trimester was previously studied by Guelfi et al. [87], Sharafeldeen et al. [88], Tu et al. [89] and Francis et al. [90]. All these studies confirmed the correlation between higher levels of FABP4 and the risk of GDM in the first trimester. Differently from Jin et al. [86], Guelfi et al. did not find any difference in FABP4 levels in the second trimester between controls and women who developed GDM [87]. In the third trimester, FABP4 levels were still higher in the GDM patients compared with controls [91], but one week before delivery, this difference seemed to recede, probably because of the increased insulin resistance that is also present in women who are not affected by GDM in late pregnancy [92].

### 2.9. HTRA-1 (High Temperature Requirement Protease A-1)

HTRA-1 is a protein normally secreted by the placenta [93]. It has serine-protease activity and is involved in several biological processes [94,95,96,97,98,99]. Its expression increases from the first to the third trimester during pregnancy [99]. HTRA-1 has a fundamental role in promoting the invasion and migration of placental cells and is a key regulator of angiogenesis [100].

Recently, Tossetta et al. studied the role of HTRA1 in an in vitro human placental model. They found that HTRA1 expression was increased in an oxidative stress condition, which is typical in women who developed late PE. Moreover, the HTRA1 contribution in cell migration and invasion was shown, underlining the possible role of HTRA1 in placental development and, consequently, in PE onset [101]. The correlation between this protein and adverse pregnancy outcomes has recently emerged. In 2019, Gesuita et al. analyzed a cohort of 158 women (14 women that developed late PE) and found that the HTRA1 levels were higher at 12 weeks in women who developed PE [102]. Moreover, in 2022, the association between this marker and GDM risk was also evaluated. HTRA1 levels were significantly higher in the first trimester in women who developed GDM. This difference was also found in the third trimester between healthy pregnant women [1.9 ng/mL (IQR: 0.8; 4.0)] and women affected by GDM [4.3 ng/mL (IQR: 3.4; 6.0)] who had higher HTRA1 levels [103]. Finally, HTRA1 levels also seem to be linked to an increased risk of preterm birth. In 2020, Giannubilo et al. found that the probability of preterm birth increased significantly with an increased HTRA1 concentration [104]. This biomarker may have a key predictive role in complicated pregnancy, but it should be combined with other markers since it cannot discriminate between pregnancy complications.

### 2.10. CD93 (Cluster of Differentiation-93)

CD93 is a glycoprotein secreted by endothelial cells; this protein has an anti-inflammatory and proangiogenic role [105]. It was demonstrated that CD93 is expressed in fetal vessels and that its expression is higher in the first trimester than in the third trimester. This characteristic may suggest the role of CD93 in modulation endothelial cell proliferation [106]. In this scenario, it appears that CD93 level changes may reflect an impairment in endothelial cell migration; in angiogenesis; and, consequently, in placentation. Recently, Piani et al. analyzed CD93 levels in the first trimester in 34 women who developed late PE and in 49 healthy women (111.8 ± 24.4 vs. 137.5 ± 22.3 ng/mL) [107]. This was the first study that analyzed the role of CD93 in the prediction of PE and found that CD93 levels were lower in women who developed PE compared with controls [107]. These results suggest a potential role of CD93 in predicting PE in the first trimester.

### 2.11. Gal-3 (Galectin-3)

Gal-3 has a potential role as a biomarker in cardiovascular diseases, in particular, in heart failure [108]. Moreover, this protein has a key role in inflammation, cell migration and proliferation, and angiogenesis [109]. Gal-3 binds strongly to advanced glycation end products and interacts with cell adhesion molecules, leading to the production of free reactive radicals and causing endothelial dysfunction [110].

Several studies already showed the correlation between Gal-3 and hyperglycemia [111,112,113]. Moreover, Gal-3 expression is altered in women affected by GDM [114] and IUGR [115].

In 2020, Talmor-Balkan et al. studied Gal-3 levels in the first trimester in 60 women: 24 later developed gestational diabetes and 36 were healthy controls. It was found that the Gal-3 levels were significantly higher in women who later developed GDM. Moreover, a significant difference in Gal-3 levels was also found in the third trimester [110]. Recently, Deng et al. studied Gal-3 expression in 190 women, in particular, 77 pregnant women who later developed GDM and 113 healthy pregnant women. It was demonstrated that the Gal-3 levels were significantly higher in the pregnant women affected by GDM in both the first (7.29 vs. 6.90 ng/mL) and second trimesters (7.41 vs. 6.95 ng/mL); however, in the third trimester, there was no difference [116]. These findings should be further investigated to validate the role of Gal-3 as a predictive biomarker of GDM.

### 2.12. Fibronectin

Fibronectin plays multiple roles, including cell adhesion, growth, migration and differentiation. It is synthesized by endothelial cells, fibroblasts and smooth muscle cells [117]. Fibronectin is considered a reliable indicator protein for endothelial function and related pathological conditions when measured in plasma. Elevated levels of circulating fibronectin have been observed in various metabolic syndromes linked to endothelial dysfunction, such as diabetes [118]. Changes in the serum or plasma levels of cellular fibronectin may reflect alterations in the extracellular matrix and vessel wall damage in diabetic patients [119].

Alanen et al. analyzed the first trimester maternal serum fibronectin levels in 19 women who developed GDM and in 59 control women with normal pregnancy outcomes. Maternal serum fibronectin levels were significantly lower in the GDM group compared with the control group (224.2 μg/mL, IQR 156.8–270.6 μg/mL vs. 264.8 μg/mL, IQR 224.6–330.6 μg/mL) [120]. This result should be confirmed with other studies that consider a larger sample size.

## 3. Non-Protein Placental Biomarkers

The development of RNA profiling technologies has expanded the catalog of RNA types and led to a greater appreciation of their biological functions. Several RNA molecules that lack the ability to encode proteins but play a key role in various cellular processes were identified. These RNA transcripts are commonly referred to as non-coding RNAs (ncRNAs). It was observed that approximately three quarters of the human genome is transcribed into ncRNAs, while protein-coding genes account for only 1–2% of the genome [121,122].

ncRNAs are classified according to the length of the nucleotides: small RNAs, with a nucleotide length of less than 200, and long RNAs, with a nucleotide length of greater than 200 [123]. MicroRNAs (miRNAs), piwi RNAs (piRNAs) and small interfering RNAs (siRNAs) are part of the former category [124]. Their main function is to silence gene transcription. Conversely, long non-coding RNAs (lncRNAs) are involved in various biological processes, such as imprinting, epigenetic modulation and transcriptional regulation [122].

Several studies focused on ncRNA levels in the blood plasma of patients who develop pregnancy complications, particularly in the case of placental diseases [123].

Table 2 will summarize non-protein placental biomarkers.

### 3.1. miRNA

While a variety of ncRNAs were identified as significant regulators of gene expression, research on placental ncRNAs has predominantly focused on miRNAs [106]. In fact, miRNAs are found in various biological fluids, such as blood and urine, in the form of complexes with lipoproteins or proteins [125]. This makes them highly stable and resistant to degradation [126]. It was shown that even freezing and thawing cycles do not alter the miRNA content of serum. Circulating miRNAs are therefore of great interest as biomarkers because they are easier to measure [127].

In particular, microRNAs play a key role in trophoblast cell proliferation, thereby regulating placental development [128]. Several studies showed altered expression of certain miRNAs by the placenta of women who developed conditions such as PE [129,130]. The detection of such altered placental markers in the first trimester of pregnancy could help to prevent or treat these serious placental disorders at an early stage.

A recent systematic review identified the main circulating miRNAs that could be considered as diagnostic markers in the first trimester for major placental diseases [131]. A total of 118 miRNAs studied in the literature were considered, and of these, 7 miRNAs were shown to be associated with these diseases. In detail, increases in miR-125b [132,133,134], miR-518b [135,136,137] and miR-628-3p [138,139] values in the first trimester were associated with PE. An increased value of miR-125b appears to be evident during the entire pregnancy in patients with PE, although the increase is more pronounced in the first trimester [134]. As for miR-628-3p, it is upregulated at 20 gestational weeks in women with PE compared with healthy controls [138].

An altered expression of miR-365a-3p [132,138] was associated with PE but its value was shown to be increased in one study and decreased in another.

miR-518b and miR-520h have also been associated with the development of PE in association with gestational hypertension. They are part of the chromosome 19 miR cluster (C19MC). Also, in this case, their values increase in the first trimester [135,136]. Moreover, Miura et al. [140] demonstrated increased levels of C19MC microRNAs, including miR-518b and miR-520h, in the maternal plasma of patients with established severe PE between 27 and 34 gestational weeks.

As for miR-374a-5p and miR-191-5p, their increased levels were found in the first trimester in the blood of patients who developed PE, growth restriction and preterm birth [132,141,142].

Several studies focused on the predictivity of the levels of different miRNAs in the first trimester regarding gestational diabetes (GDM). Juchnicka et al. [143] examined 800 miRNAs and studied their concentrations in maternal blood during the first trimester to investigate a possible correlation with the development of GDM. Their results show the upregulation of miR-16-5p, miR-142-3p and miR-144-3p in the first trimester in patients that developed GDM.

Regarding miR-16-5p, this is one of the most powerful regulatory molecules in the insulin pathway. The target genes of miR-16-5p encode proteins that are essential for proper insulin signaling. Their downregulation may be responsible for metabolic disorders, such as insulin resistance [144,145]. There are also other studies in the literature that show high circulating levels of miR-16-5p in the second trimester of a pregnancy complicated by GDM [146,147].

As for miR-142-3p, this has the forkhead box O1 (FOXO1) protein as its target gene. It has several functions, including controlling glycogenolysis and gluconeogenesis and regulating B-cell differentiation [148]. miR-142-3p was also investigated in other previous studies [149], but none of them assessed its plasma concentration in humans.

Concerning miR-144-3p, previous articles show its upregulation in the peripheral blood of patients with type 1, type 2 diabetes mellitus and GDM [149,150].

### 3.2. Long Non-Coding RNA (lncRNAs)

lncRNAs have attracted a great deal of interest because, in addition to regulating biological processes, such as imprinting, the cell cycle and angiogenesis, they have also been implicated in the development of various diseases, including cardiovascular diseases and cancer [151,152,153]. Recent studies focused on their role in the pathogenesis of placental disorders, such as PE, by demonstrating aberrant lncRNA production in the placental tissue of these patients. Moradi et al. investigated 14 dysregulated lncRNAs in patients with PE and validated them by qPCR analysis [154]. Lots of current studies are limited to detecting an upregulation or downregulation of lncRNAs in placental tissues after delivery in patients with PE compared with patients with a normal pregnancy [154]. This suggests a key role of lncRNAs in the pathophysiology of PE. However, there are currently no studies that have identified lncRNAs as first trimester diagnostic biomarkers to support the early diagnosis of placental disorders. Peñailillo et al. [155] recently attempted to address this gap in the literature. Their study showed that lncRNA LOC101927355 is decreased in the placenta and maternal plasma at the time of delivery in patients with PE, for which it could represent a new potential biomarker. However, this should be validated with plasma samples collected during the first half of pregnancy before the development of pre-eclamptic symptoms.

Regarding gestational diabetes, the dysregulation of major circulating or placental lncRNAs appears to influence insulin resistance due to a dysfunction of pancreatic β cells [156]. Several studies evaluated the aberrant production of certain types of lncRNAs in patients with GDM. A recent study by Jiang et al. [157] evaluated the values of circulating lncRNAs NONHSAT054669.2 and ENST00000525337 in the first, second and third trimesters of women with GDM and women with normal glucose tolerance (NGT). These makers were more highly expressed in the plasma of the case group in the first and second trimesters. Thus, this makes them interesting early diagnostic markers of this pathology. In the literature, other studies focused on other types of circulating lncRNAs. Su et al. [158] demonstrated a significant increase in the lncRNA HOTAIR in GDM patients, but the blood sample was taken from a group of women already diagnosed with diabetes and it is therefore not possible to know whether the same result would have been obtained in the first trimester. Moreover, Zhang et al. [159] showed an increased plasma concentration of the lncRNA MALAT1 in women with GDM, but plasma samples were obtained at 36–40 weeks of pregnancy. As in the previous study, the role of MALAT1 as a predictive biomarker in the first trimester was not investigated.

### 3.3. ADMA (Asymmetric Dimethylarginine)

ADMA is an amino acid and is an inhibitor of nitric oxide (NO) synthesis. In physiologic conditions, ADMA concentrations in the first trimester fall below pre-pregnant concentrations [160]. The reason is, probably, the increased levels of NO, which determine decreased maternal blood pressure in the first trimester [161]. NO mediates vasodilation and contributes to low fetoplacental vascular resistance [162].

Several researchers studied whether increased levels of ADMA, and consequently, decreased levels of NO, were linked to an increased risk of PE. Different studies show contrasting results. In 2013, Khalil et al. demonstrated that there was no difference between PE and controls for ADMA levels in 375 pregnant women (300 controls and 75 women who developed PE) [163]. In 2015, a prospective case–control study conducted on 740 pregnant women in the first trimester evaluated the association between ADMA and PE. Forty women developed late PE. It was found that ADMA levels were significantly higher in women who developed PE compared with controls (0.86 ± 0.16 vs. 0.68 ± 0.20 μM, *p* < 0.001) [164]. Due to these contrasting results, other studies with a bigger cohort of patients are required to evaluate ADMA’s predictive role in PE.

## 4. Discussion and Conclusions

Normal placental development is a tightly regulated process that has a key role in the pregnancy outcome and, when this process is impaired, can lead to several pregnancy complications [165,166,167,168]. Several protein and non-protein biomarkers have been correlated with some adverse pregnancy outcomes, such as GDM, PE or IUGR. In this review, we focused our attention on the principal serum biomarkers, showing that for some biomarkers, the correlation with pregnancy outcomes is not so clear since there are still a lot of contrasting data.

Studying principal adverse obstetrical outcomes, different researchers found that the pathophysiological mechanisms include inflammatory response, oxidative stress and endothelial dysfunction. These are associated with an increased hypoxia of the placenta, and consequently, altered placental function [169]. It emerges that many pregnancy complications share the same mechanisms, as shown by the levels of afamin, HTRA-1 and FABP4 in the first trimester [170]. It is already known that the PE incidence is increased in women affected by GDM and vice versa; the link between PE and GDM may probably be explained by shared pathophysiological mechanisms [170]. Recently, Artemieva et al. analyzed the morphofunctional changes in the placenta in women affected by GDM and PE, underlining a similar placental damage both in GDM and PE [171]. In particular, the increased inflammatory response seems to be similar in GDM and PE: neutrophils are overactive and contribute to worsening the inflammation [172]. Moreover, oxidative stress plays a key role in GDM and PE [169]. Placental dysfunction mechanisms in GDM and PE are similar: the inflammatory response and the oxidative stress seem to have a key role, both in GDM and in PE. This scenario may explain why some placental biomarkers, such as HTRA-1 or afamin, are altered in both GDM and PE. However, the exact mechanisms underlying the association between placental dysfunction in GDM and in PE are still unclear, and thus, further studies are required in order to better clarify this point.

As for protein biomarkers, non-protein biomarkers also have a key role in predicting adverse obstetrical outcomes. As reported before, the role of miRNA and lncRNA in placental development and angiogenesis is crucial. In this scenario, altered levels of non-protein biomarkers may reflect placental dysfunction.

In summary, the placenta is a vital organ; its impaired function is responsible for certain obstetric pathologies, including GDM or preeclampsia. In this context, the importance of identifying early markers of placental damage that can predict the risk of developing an obstetric disorder becomes evident.

The creation of predictive models requires studies about the association of different biomarkers and the principal pregnancy complications. In this scenario, it appears that we are still far from finding a predictive model in the first trimester for at-risk pregnancies. This model would help clinicians to do the following:

- Reduce the risk of developing a pregnancy complication;
- Detect the pregnancy complication earlier;
- Reduce the severity of the complication;
- Improve the management and the therapy of the complication.

Thus, further studies are needed in order to find predictive models to be used in the first trimester.

## Figures and Tables

**Table 1:** Protein placental biomarkers in first trimester.

Protein Marker	PLGF	PAPP-A	BetaHCG	PP13	ADAM-12	SHBG	Afamin	FABP4	HTRA-1	CD93	GAL-3	Fibronectin
PE	↓	↓	Contrasting data	Contrasting data	Contrasting data	No information	↑	↑	↑	↓	No information	No information
GDM	No information	No information	Contrasting data	↓	No information	Contrasting data	↑	↑	↑	No information	↑	↓

**Table 2:** Non-protein placental biomarkers in first trimester.

Non-Protein Marker	miR-125b	miR-518b	miR-628-3p	miR-365a-3p	miR-520h	miR-374a-5p	miR-191-5p	miR-16-5p	miR-142-3p	miR-144-3p	lncRNAs NONHSAT054669.2	lncRNAs ENST00000525337	ADMA
PE	↑	↑	↑	Contrasting data	↑	↑	↑	No information	No information	No information	No information	No information	Contrasting data
GDM	No information	No information	No information	No information	No information	No information	No information	↑	↑	↑	↑	↑	No information

## References

[1] Konkel L. (2016). Lasting Impact of an Ephemeral Organ: The Role of the Placenta in Fetal Programming. Environ. Heal. Perspect..

[2] Burton G.J., Charnock-Jones D.S., Jauniaux E. (2009). Regulation of vascular growth and function in the human placenta. Reproduction.

[3] Burton G.J., Fowden A.L. (2015). The placenta: A multifaceted, transient organ. Philos. Trans. R. Soc. B Biol. Sci..

[4] Napso T., Yong H.E.J., Lopez-Tello J., Sferruzzi-Perri A.N. (2018). The Role of Placental Hormones in Mediating Maternal Adaptations to Support Pregnancy and Lactation. Front. Physiol..

[5] Fisher S.J. (2015). Why is placentation abnormal in preeclampsia?. Am. J. Obstet. Gynecol..

[6] Hustin J., Jauniaux E., Schaaps J. (1990). Histological study of the materno-embryonic interface in spontaneous abortion. Placenta.

[7] Romero R., Kusanovic J.P., Chaiworapongsa T., Hassan S.S. (2011). Placental bed disorders in preterm labor, preterm PROM, spontaneous abortion and abruptio placentae. Best Pract. Res. Clin. Obstet. Gynaecol..

[8] Pijnenborg R., Anthony J., Davey D.A., Rees A., Tiltman A., Vercruysse L., Van Assche A. (1991). Placental bed spiral arteries in the hypertensive disorders of pregnancy. BJOG Int. J. Obstet. Gynaecol..

[9] Olmos-Ortiz A., Flores-Espinosa P., Díaz L., Velázquez P., Ramírez-Isarraraz C., Zaga-Clavellina V. (2021). Immunoendocrine Dysregulation during Gestational Diabetes Mellitus: The Central Role of the Placenta. Int. J. Mol. Sci..

[10] Alqudah A., Eastwood K.-A., Jerotic D., Todd N., Hoch D., McNally R., Obradovic D., Dugalic S., Hunter A.J., Holmes V.A. (2021). FKBPL and SIRT-1 Are Downregulated by Diabetes in Pregnancy Impacting on Angiogenesis and Endothelial Function. Front. Endocrinol..

[11] Carrasco-Wong I., Moller A., Giachini F.R., Lima V.V., Toledo F., Stojanova J., Sobrevia L., Martín S.S. (2020). Placental structure in gestational diabetes mellitus. Biochim. Biophys. Acta (BBA)—Mol. Basis Dis..

[12] Melchiorre K., Giorgione V., Thilaganathan B. (2021). The placenta and preeclampsia: Villain or victim?. Am. J. Obstet. Gynecol..

[13] Jena M.K., Sharma N.R., Petitt M., Maulik D., Nayak N.R. (2020). Pathogenesis of Preeclampsia and Therapeutic Approaches Targeting the Placenta. Biomolecules.

[14] Tossetta G., Fantone S., Piani F., Crescimanno C., Ciavattini A., Giannubilo S.R., Marzioni D. (2023). Modulation of NRF2/KEAP1 Signaling in Preeclampsia. Cells.

[15] Aplin J.D., Myers J.E., Timms K., Westwood M. (2020). Tracking placental development in health and disease. Nat. Rev. Endocrinol..

[16] Ilekis J.V., Tsilou E., Fisher S., Abrahams V.M., Soares M.J., Cross J.C., Bidwell G. (2016). Placental origins of adverse preg-nancy out-comes: Potential molecular targets: An executive workshop summary of the eunice Kennedy Shriver Na-tional Institute of child Health and human development. Am. J. Obstet. Gynecol..

[17] Jansen C.H., Kastelein A.W., Kleinrouweler C.E., Van Leeuwen E., De Jong K.H., Pajkrt E., Van Noorden C.J. (2020). Devel-opment of placental abnormalities in location and anatomy. Acta Obstet. Gynecol. Scand..

[18] American College of Obstetricians and Gynecologists (2013). Task Force on Hypertension in Pregnancy. Hypertension in pregnancy. Report of the American College of Obstetricians and Gynecologists’ Task Force on Hypertension in Pregnancy. Obstet. Gynecol..

[19] Aronson J.K., Ferner R.E. (2017). Biomarkers—A General Review. Curr. Protoc. Pharmacol..

[20] Lim J.H., Kim S.Y., Park S.Y., Yang J.H., Kim M.Y., Ryu H.M. (2008). Effective Prediction of Preeclampsia by a Combined Ratio of Angiogenesis-Related Factors. Obstet. Gynecol..

[21] Herraiz I., Llurba E., Verlohren S., Galindo A. (2018). Update on the diagnosis and prognosis of preeclampsia with the aid of the sFlt-1/PlGF ratio in singleton pregnancies. Fetal Diagn. Ther..

[22] Lecarpentier E., Zsengeller Z.K., Salahuddin S., Covarrubias A.E., Lo A., Haddad B., Thadhani R.I., Karumanchi S.A. (2020). Total Versus Free Placental Growth Factor Levels in the Pathogenesis of Preeclampsia. Hypertension.

[23] Allen R.E., Rogozinska E., Cleverly K., Aquilina J., Thangaratinam S. (2014). Abnormal blood biomarkers in early pregnancy are as-sociated with preeclampsia: A meta-analysis. Eur. J. Obstet. Gynecol. Reprod. Biol..

[24] Herraiz I., Dröge L.A., Gómez-Montes E., Henrich W., Galindo A., Verlohren S. (2014). Characterization of the soluble fms-like tyro-sine kinase-1 to placental growth factor ratio in pregnancies complicated by fetal growth restriction. Obstet. Gynecol..

[25] Sovio U., Gaccioli F., Cook E., Charnock-Jones D.S., Smith G.C. (2021). Slowing of fetal growth and elevated maternal serum sFLT1:PlGF are associated with early term spontaneous labor. Am. J. Obstet. Gynecol..

[26] Chappell L.C., Duckworth S., Seed P.T., Griffin M., Myers J., Mackillop L., Simpson N., Waugh J., Anumba D., Kenny L.C. (2013). Diagnostic accuracy of placental growth factor in women with suspected preeclampsia: A prospective multicenter study. Circulation.

[27] The Fetal Medicine Foundation Risk Assessment: Risk for Preeclampsia. https://fetalmedicine.org/research/assess/preeclampsia/first-trimester.

[28] Levine R.J., Maynard S.E., Qian C., Lim K.-H., England L.J., Yu K.F., Schisterman E.F., Thadhani R., Sachs B.P., Epstein F.H. (2004). Circulating Angiogenic Factors and the Risk of Preeclampsia. N. Engl. J. Med..

[29] Cerdeira A.S., O’Sullivan J., Ohuma E.O., James T., Papageorghiou A.T., Knight M., Vatish M. (2021). Performance of soluble fms-like tyrosine kinase-1-to-placental growth factor ratio of ≥85 for ruling in preeclampsia within 4 weeks. Am. J. Obstet. Gynecol..

[30] NICE PlGF-Based Testing to Help Diagnose Suspected Preeclampsia (Triage PlGF Test, Elecsys Immunoassay sFlt-1/PlGF ra-Tio, DELFIA Xpress PlGF 1-2-3 Test, and BRAHMS sFlt-1 Kryptor/BRAHMS PlGF Plus Kryptor PE Ratio). Diagnostics Guidance [DG23]. https://www.nice.org.uk/guidance/dg23/.

[31] Stepan H., Galindo A., Hund M., Schlembach D., Sillman J., Surbek D., Vatish M. (2023). Clinical utility of sFlt-1 and PlGF in screening, prediction, diagnosis and monitoring of pre-eclampsia and fetal growth restriction. Ultrasound Obstet. Gynecol..

[32] Alldred S.K., Takwoingi Y., Guo B., Pennant M., Deeks J.J., Neilson J.P., Cochrane Pregnancy and Childbirth Group (1996). First trimester ultrasound tests alone or in combina-tion with first trimester serum tests for Down’s syndrome screening. Cochrane Database Syst. Rev..

[33] Conover C.A., Oxvig C. (2023). The Pregnancy-Associated Plasma Protein-A (PAPP-A) Story. Endocr. Rev..

[34] Fruscalzo A., Cividino A., Rossetti E., Maurigh A., Londero A.P., Driul L. (2020). First trimester PAPP-A serum levels and long-term metabolic outcome of mothers and their offspring. Sci. Rep..

[35] Sun X.-W., Li X.-H., Zhang C., Meng F.-Q., Xing Y.-G., Ding Y. (2021). Correlation analysis of serum placental growth factor, pregnancy-related plasma protein-A and disease severity in patients with hypertensive disorder in pregnancy. Eur. Rev. Med. Pharmacol. Sci..

[36] Shahsavandi E., Movahedi M., Khanjani S., Shahshahan Z., Hajihashemi M., Farahbod F. (2023). Evaluation of the relationship between pregnancy-associated plasma protein A (PAPP-A) and pregnancy outcomes. Adv. Biomed. Res..

[37] Gagnon A., Wilson R.D., Audibert F., Allen V.M., Blight C., Brock J.A., Wyatt P. (2008). Obstetrical complications assoicated with abnormal maternal serum marker analytes. J. Obstet. Gynaecol. Can..

[38] Balkans G., Caglar T. (2023). Elevated first-trimester PAPP-A is a marker in high-risk pregnancies with an increased risk of placenta accreta in predicting adverse outcomes. Eur. Rev. Med. Pharmacol. Sci..

[39] Xu H., Liu X., Li P., Du W., Han Q. (2019). Association of pregnancy-associated plasma protein A and vascular endothelial growth fac-tor with pregnancy-induced hypertension. Exp. Ther. Med..

[40] Cole L.A. (2010). Biological functions of hCG and hCG-related molecules. Reprod. Biol. Endocrinol..

[41] Jing G., Yao J., Dang Y., Liang W., Xie L., Chen J., Li Z. (2020). The role of β-HCG and VEGF-MEK/ERK signaling pathway in villi angiogenesis in patients with missed abortion. Placenta.

[42] Berndt S., D’Hauterive S.P., Blacher S., Péqueux C., Lorquet S., Munaut C., Applanat M., Hervé M.A., Lamandé N., Corvol P. (2006). Angiogenic activity of human chorionic gonadotropin through LH receptor activation on endothelial and epithelial cells of the endometrium. FASEB J..

[43] Reisinger K., Baal N., McKinnon T., Münstedt K., Zygmunt M. (2007). The gonadotropins: Tissue-specific angiogenic factors?. Mol. Cell. Endocrinol..

[44] Younesi S., Eslamian L., Khalafi N., Amin M.M.T., Saadati P., Jamali S., Balvayeh P., Modarressi M.-H., Savad S., Amidi S. (2023). Extreme βHCG levels in first trimester screening are risk factors for adverse maternal and fetal outcomes. Sci. Rep..

[45] Visconti F., Quaresima P., Chiefari E., Caroleo P., Arcidiacono B., Puccio L., Mirabelli M., Foti D.P., Di Carlo C., Vero R. (2019). First Trimester Combined Test (FTCT) as a Predictor of Gestational Diabetes Mellitus. Int. J. Environ. Res. Public Health.

[46] Husslein H., Lausegger F., Leipold H., Worda C. (2012). Association between pregnancy-associated plasma protein-A and gestational diabetes requiring insulin treatment at 11–14 weeks of gestation. J. Matern. Neonatal Med..

[47] Zhang X., Huangfu Z., Shi F., Xiao Z. (2021). Predictive Performance of Serum β-hCG MoM Levels for Preeclampsia Screening: A Meta-Analysis. Front. Endocrinol..

[48] Huppertz B., Sammar M., Chefetz I., Neumaier-Wagner P., Bartz C., Meiri H. (2008). Longitudinal Determination of Serum Placental Protein 13 during Development of Preeclampsia. Fetal Diagn. Ther..

[49] Than N.G., Balogh A., Romero R., Kárpáti E., Erez O., Szilágyi A., Kovalszky I., Sammar M., Gizurarson S., Matkó J. (2014). Placental Protein 13 (PP13)—A Placental Immunoregulatory Galectin Protecting Pregnancy. Front. Immunol..

[50] Gadde R., Cd D., Sheela S. (2018). Placental protein 13: An important biological protein in preeclampsia. J. Circ. Biomark..

[51] Vasilache I.-A., Carauleanu A., Socolov D., Matasariu R., Pavaleanu I., Nemescu D. (2022). Predictive performance of first trimester serum galectin-13/PP-13 in preeclampsia screening: A systematic review and meta-analysis. Exp. Ther. Med..

[52] Tenenbaum-Gavish K., Sharabi-Nov A., Binyamin D., Møller H.J., Danon D., Rothman L., Hadar E., Idelson A., Vogel I., Koren O. (2020). First trimester biomarkers for prediction of gestational diabetes mellitus. Placenta.

[53] Biadasiewicz K., Fock V., Dekan S., Proestling K., Velicky P., Haider S., Knöfler M., Fröhlich C., Pollheimer J. (2014). Extravillous Trophoblast-Associated ADAM12 Exerts Pro-Invasive Properties, Including Induction of Integrin Beta 1-Mediated Cellular Spreading1. Biol. Reprod..

[54] Ratnik K., Rull K., Aasmets O., Kikas T., Hanson E., Kisand K., Fischer K., Laan M. (2022). Novel Early Pregnancy Multimarker Screening Test for Preeclampsia Risk Prediction. Front. Cardiovasc. Med..

[55] Yu N., Cui H., Chen X., Chang Y. (2017). First trimester maternal serum analytes and second trimester uterine artery Doppler in the prediction of preeclampsia and fetal growth restriction. Taiwan. J. Obstet. Gynecol..

[56] Karagiannis G., Akolekar R., Sarquis R., Wright D., Nicolaides K.H. (2011). Prediction of small-for-gestation neonates from bio-physical and biochemical markers at 11–13 weeks. Fetal Diagn. Ther..

[57] Cowans N.J., Spencer K. (2007). First-trimester ADAM12 and PAPP-A as markers for intrauterine fetal growth restriction through their roles in the insulin-like growth factor system. Prenat. Diagn..

[58] Andres F., Wong G.P., Walker S.P., MacDonald T.M., Keenan E., Cannon P., Nguyen T.-V., Hannan N.J., Tong S., Kaitu’U-Lino T.J. (2022). A disintegrin and metalloproteinase 12 (ADAM12) is reduced at 36 weeks’ gestation in pregnancies destined to deliver small for gestational age infants. Placenta.

[59] Kasimis C., Evangelinakis N., Rotas M., Georgitsi M., Pelekanos N., Kassanos D. (2016). Predictive value of biochemical marker AD-AM-12 at first trimester of pregnancy for hypertension and intrauterine growth restriction. Clin. Exp. Obstet. Gynecol..

[60] Zamarian A.C.P., Júnior E.A., Daher S., Rolo L.C., Moron A.F., Nardozza L.M.M. (2016). Evaluation of biochemical markers combined with uterine artery Doppler parameters in fetuses with growth restriction: A case–control study. Arch. Gynecol. Obstet..

[61] Sherif K., Kushner H., Falkner B.E. (1998). Sex hormone-binding globulin and insulin resistance in African-American women. Metabolism.

[62] Haffner S.M., Valdez R.A., Morales P.A., Hazuda H.P., Stern M.P. (1993). Decreased sex hormone-binding globulin predicts noninsulin dependent diabetes mellitus in wom-en but not in men. Endocr. Soc..

[63] Thaler M.A., Seifert-Klauss V., Luppa P.B. (2015). The biomarker sex hormone-binding globulin—From established applications to emerging trends in clinical medicine. Best Pract. Res. Clin. Endocrinol. Metab..

[64] Ding E.L., Song Y., Manson J.E., Hunter D.J., Lee C.C., Rifai N., Liu S. (2009). Sex hormone-binding globulin and risk of type 2 diabe-tes in women and men. N. Engl. J. Med..

[65] Le T.N., Nestler J.E., Strauss J.F., Wickham E.P. (2012). Sex hormone-binding globulin and type 2 diabetes mellitus. Trends Endocrinol. Metab..

[66] Basil B., Oghagbon E.K., Mba I.N., Adebisi S.A., Agudi C.C. (2022). First trimester sex hormone-binding globulin predicts gestational diabetes mellitus in a population of Nigerian women. J. Obstet. Gynaecol..

[67] Zhang T., Du T., Li W., Yang S., Liang W. (2018). Sex hormone-binding globulin levels during the first trimester may predict gestational diabetes mellitus development. Biomark. Med..

[68] Correa P.J., Venegas P., Palmeiro Y., Albers D., Rice G., Roa J., Cortez J., Monckeberg M., Schepeler M., Osorio E. (2018). First trimester prediction of gestational diabetes mellitus using plasma biomarkers: A case-control study. Jpme.

[69] Liu W., Huang Z., Tang S., Zhang Z., Yu Q., He J. (2021). Changes of Serum Sex Hormone-Binding Globulin, Homocysteine, and Hypersensitive CRP Levels during Pregnancy and Their Relationship with Gestational Diabetes Mellitus. Gynecol. Obstet. Investig..

[70] Faal S., Abedi P., Jahanfar S., Ndeke J.M., Mohaghegh Z., Sharifipour F., Zahedian M. (2019). Sex hormone binding globulin for prediction of gestational diabetes mellitus in pre-conception and pregnancy: A systematic review. Diabetes Res. Clin. Pract..

[71] Berggren E.K., Boggess K.A., Mathew L., Culhane J. (2016). First Trimester Maternal Glycated Hemoglobin and Sex Hormone-Binding Globulin Do Not Predict Third Trimester Glucose Intolerance of Pregnancy. Reprod. Sci..

[72] Voegele A.F., Jerković L., Wellenzohn B., Eller P., Kronenberg F., Liedl K.R., Dieplinger H. (2002). Characterization of the Vitamin E-Binding Properties of Human Plasma Afamin. Biochemistry.

[73] Niki E., Traber M.G. (2012). A History of Vitamin E. Ann. Nutr. Metab..

[74] Koeninger A., Enekwe A., Mach P., Andrikos D., Schmidt B., Frank M., Birdir C., Kimmig R., Gellhaus A., Dieplinger H. (2018). Afamin: An early predictor of preeclampsia. Arch. Gynecol. Obstet..

[75] Köninger A., Mathan A., Mach P., Frank M., Schmidt B., Schleussner E., Kimmig R., Gellhaus A., Dieplinger H. (2018). Is Afamin a novel biomarker for gestational diabetes mellitus? A pilot study. Reprod. Biol. Endocrinol..

[76] Tramontana A., Dieplinger B., Stangl G., Hafner E., Dieplinger H. (2018). First trimester serum afamin concentrations are associated with the development of pre-eclampsia and gestational diabetes mellitus in pregnant women. Clin. Chim. Acta.

[77] Gülücü S., Çelik S., Unver G. (2023). Evaluation of first- and third-trimester afamin levels in preeclampsia. Rev. Assoc. Med. Bras..

[78] Çalışkan C.S., Celik S., Avcı B. (2020). Is afamin a potential early biomarker for subsequent development of preeclampsia? A nested case–control study. J. Matern. Neonatal Med..

[79] Yuan Y., He W., Fan X., Liang J., Cao Z., Li L. (2023). Serum afamin levels in predicting gestational diabetes mellitus and preeclampsia: A systematic review and meta-analysis. Front. Endocrinol..

[80] Furuhashi M., Saitoh S., Shimamoto K., Miura T. (2015). Fatty Acid-Binding Protein 4 (FABP4): Pathophysiological insights and po-tent clinical biomarker of metabolic and cardiovascular diseases. Clin. Med. Insights Cardiol..

[81] Ron I., Mdah R., Zemet R., Ulman R.Y., Rathaus M., Brandt B., Mazaki-Tovi S., Hemi R., Barhod E., Tirosh A. (2023). Adipose tissue-derived FABP4 mediates glucagon-stimulated hepatic glucose production in gestational diabetes. Diabetes Obes. Metab..

[82] Na Ge X., Bastan I., Dileepan M., Greenberg Y., Gil Ha S., Steen K.A., Bernlohr D.A., Rao S.P., Sriramarao P. (2018). FABP4 regulates eosinophil recruitment and activation in allergic airway inflammation. Am. J. Physiol. Cell. Mol. Physiol..

[83] Shi Y., Wang C.-C., Wu L., Zhang Y., Xu A., Wang Y. (2023). Pathophysiological Insight into Fatty Acid-Binding Protein-4: Multifaced Roles in Reproduction, Pregnancy, and Offspring Health. Int. J. Mol. Sci..

[84] Paiboonborirak C., Phupong V. (2024). Serum fatty acid binding protein 4 and Doppler of uterine artery ultrasound in the first trimester for the prediction of preeclampsia. Hypertens. Res..

[85] Scifres C.M., Catov J.M., Simhan H. (2012). Maternal Serum Fatty Acid Binding Protein 4 (FABP4) and the Development of Preeclampsia. J. Clin. Endocrinol. Metab..

[86] Jin C., Lin L., Han N., Zhao Z., Xu X., Luo S., Liu J., Wang H. (2021). Risk of Gestational Diabetes Mellitus in relation to Plasma Concentrations of Fatty Acid-Binding Protein 4: A Nested Case-Control Study in China. J. Diabetes Res..

[87] Guelfi K.J., Ong M.J., Li S., Wallman K.E., Doherty D.A., Fournier P.A., Newnham J.P., Keelan J.A. (2017). Maternal circulating adipokine profile and insulin resistance in women at high risk of developing gestational diabetes mellitus. Metabolism.

[88] Sharafeldeen A. (2018). Serum adropin and adipose fatty-acid binding protein at 6th week of pregnancy are significant predictors for development of insulin resistance at the 24th week. Int. J. Adv. Res..

[89] Tu W.-J., Guo M., Shi X.-D., Cai Y., Liu Q., Fu C.-W. (2017). First-Trimester Serum Fatty Acid-Binding Protein 4 and Subsequent Gestational Diabetes Mellitus. Obstet. Gynecol..

[90] Francis E.C., Li M., Hinkle S.N., Cao Y., Chen J., Wu J., Zhu Y., Cao H., Kemper K., Rennert L. (2020). Adipokines in early and mid-pregnancy and subsequent risk of gestational diabetes: A longitudinal study in a multiracial cohort. BMJ Open Diabetes Res. Care.

[91] Zhang Y., Zhang H., Lu J., Zheng S., Long T., Li Y., Wu W., Wang F. (2016). Changes in serum adipocyte fatty acid-binding protein in women with gestational diabetes mellitus and normal pregnant women during mid- and late pregnancy. J. Diabetes Investig..

[92] Ortega-Senovilla H., Schaefer-Graf U., Meitzner K., Abou-Dakn M., Graf K., Kintscher U., Herrera E. (2011). Gestational Diabetes Mellitus Causes Changes in the Concentrations of Adipocyte Fatty Acid–Binding Protein and Other Adipocytokines in Cord Blood. Diabetes Care.

[93] Fantone S., Giannubilo S.R., Marzioni D., Tossetta G. (2021). HTRA family proteins in pregnancy outcome. Tissue Cell.

[94] Goteri G., Altobelli E., Tossetta G., Zizzi A., Avellini C., Licini C., Lorenzi T., Castellucci M., Ciavattini A., Marzioni D. (2015). High Temperature Requirement A1, Transforming Growth Factor Beta 1, phosphoSmad2 and Ki67 in Eutopic and Ectopic Endometrium of Women with Endometriosis. Eur. J. Histochem..

[95] Altobelli E., Latella G., Morroni M., Licini C., Tossetta G., Mazzucchelli R., Profeta V.F., Coletti G., Leocata P., Castellucci M. (2017). Low HtrA1 expression in patients with long-standing ulcerative colitis and colorectal cancer. Oncol. Rep..

[96] Tossetta G., Fantone S., Licini C., Marzioni D., Mattioli-Belmonte M. (2022). The multifaced role of HtrA1 in the development of joint and skeletal disorders. Bone.

[97] Licini C., Fantone S., Lamanna D., Tossetta G., Marzioni D., Belmonte M.M. (2024). Possible involvement of HtrA1 serine protease in the onset of osteoporotic bone extracellular matrix changes. Tissue Cell.

[98] Tossetta G., Avellini C., Licini C., Giannubilo S., Castellucci M., Marzioni D. (2016). High temperature requirement A1 and fibronectin: Two possible players in placental tissue remodelling. Eur. J. Histochem..

[99] De Luca A., De Falco M., Fedele V., Cobellis L., Mastrogiacomo A., Laforgia V., Tuduce I.L., Campioni M., Giraldi D., Paggi M.G. (2004). The Serine Protease HtrA1 Is Upregulated in the Human Placenta during Pregnancy. J. Histochem. Cytochem..

[100] Zhang L., Lim S.L., Du H., Zhang M., Kozak I., Hannum G., Wang X., Ouyang H., Hughes G., Zhao L. (2012). High Temperature Requirement Factor A1 (HTRA1) Gene Regulates Angiogenesis through Transforming Growth Factor-β Family Member Growth Differentiation Factor 6. J. Biol. Chem..

[101] Tossetta G., Fantone S., Giannubilo S.R., Ciavattini A., Senzacqua M., Frontini A., Marzioni D. (2023). HTRA1 in Placental Cell Models: A Possible Role in Preeclampsia. Curr. Issues Mol. Biol..

[102] Gesuita R., Licini C., Picchiassi E., Tarquini F., Coata G., Fantone S., Tossetta G., Ciavattini A., Castellucci M., Di Renzo G.C. (2019). Association between first trimester plasma htra1 level and subsequent preeclampsia: A possible early marker?. Pregnancy Hypertens..

[103] Tossetta G., Fantone S., Gesuita R., Di Renzo G.C., Meyyazhagan A., Tersigni C., Scambia G., Di Simone N., Marzioni D. (2022). HtrA1 in Gestational Diabetes Mellitus: A Possible Biomarker?. Diagnostics.

[104] Giannubilo S.R., Licini C., Picchiassi E., Tarquini F., Coata G., Fantone S., Tossetta G., Ciavattini A., Castellucci M., Giardina I. (2022). First trimester HtrA1 maternal plasma level and spontaneous preterm birth. J. Matern. Neonatal Med..

[105] Tossetta G., Piani F., Borghi C., Marzioni D. (2023). Role of CD93 in Health and Disease. Cells.

[106] Fantone S., Tossetta G., Di Simone N., Tersigni C., Scambia G., Marcheggiani F., Giannubilo S.R., Marzioni D. (2021). CD93 a potential player in cytotrophoblast and endothelial cell migration. Cell Tissue Res..

[107] Piani F., Tossetta G., Fantone S., Agostinis C., Di Simone N., Mandalà M., Bulla R., Marzioni D., Borghi C. (2023). First Trimester CD93 as a Novel Marker of Preeclampsia and Its Complications: A Pilot Study. High Blood Press. Cardiovasc. Prev..

[108] Andrejic O.M., Vucic R.M., Pavlovic M., McClements L., Stokanovic D., Jevtovic–Stoimenov T., Nikolic V.N. (2019). Association between Galectin-3 levels within central and peripheral venous blood, and adverse left ventricular remodelling after first acute myocardial infarction. Sci. Rep..

[109] Jia W., Kidoya H., Yamakawa D., Naito H., Takakura N. (2013). Galectin-3 Accelerates M2 Macrophage Infiltration and Angiogenesis in Tumors. Am. J. Pathol..

[110] Talmor-Barkan Y., Chezar-Azerrad C., Kruchin B., Leshem-Lev D., Levi A., Hadar E., Kornowski R., Tenenbaum-Gavish K., Porter A. (2020). Elevated galectin-3 in women with gestational diabetes mellitus, a new surrogate for cardiovascular disease in women. PLoS ONE.

[111] Yilmaz H., Cakmak M., Inan O., Darcin T., Akcay A. (2014). Increased levels of galectin-3 were associated with prediabetes and diabetes: New risk factor?. J. Endocrinol. Investig..

[112] Li P., Liu S., Lu M., Bandyopadhyay G., Oh D., Imamura T., Johnson A.M.F., Sears D., Shen Z., Cui B. (2016). Hematopoietic-Derived Galectin-3 Causes Cellular and Systemic Insulin Resistance. Cell.

[113] Darrow A.L., Shohet R.V. (2015). Galectin-3 deficiency exacerbates hyperglycemia and the endothelial response to diabetes. Cardiovasc. Diabetol..

[114] Heusler I., Biron-Shental T., Farladansky-Gershnabel S., Pasternak Y., Kidron D., Vulih-Shuitsman I., Einbinder Y., Cohen-Hagai K., Benchetrit S., Zitman-Gal T. (2021). Enhanced expression of Galectin-3 in gestational diabetes. Nutr. Metab. Cardiovasc. Dis..

[115] Freitag N., Tirado-Gonzalez I., Barrientos G., Powell K.L., Boehm-Sturm P., Koch S.P., Hecher K., Staff A.C., Arck P.C., Diemert A. (2020). Galectin-3 deficiency in pregnancy increases the risk of fetal growth restriction (FGR) via placental insufficiency. Cell Death Dis..

[116] Deng Y., Jin H., Ning J., Cui D., Zhang M., Yang H. (2023). Elevated galectin-3 levels detected in women with hyperglycemia during early and mid-pregnancy antagonizes high glucose—Induced trophoblast cells apoptosis via galectin-3/foxc1 pathway. Mol. Med..

[117] Peters J.H., Ginsberg M.H., Bohl B.P., A Sklar L., Cochrane C.G. (1986). Intravascular release of intact cellular fibronectin during oxidant-induced injury of the in vitro perfused rabbit lung. J. Clin. Investig..

[118] Ruoslahti E. (1988). FIBRONECTIN AND ITS RECEPTORS. Annu. Rev. Biochem..

[119] Lockwood C.J., Peters J.H. (1990). Increased plasma levels of ED1+ cellular fibronectin precede the clinical signs of preeclampsia. Am. J. Obstet. Gynecol..

[120] Alanen J., Appelblom H., Korpimaki T., Kouru H., Sairanen M., Gissler M., Ryynanen M., Nevalainen J. (2020). Glycosylated fibronectin as a first trimester marker for gestational diabetes. Arch. Gynecol. Obstet..

[121] Djebali S., Davis C.A., Merkel A., Dobin A., Lassmann T., Mortazavi A., Tanzer A., Lagarde J., Lin W., Schlesinger F. (2012). Landscape of transcription in human cells. Nature.

[122] Monteiro L.J., Peñailillo R., Sánchez M., Acuña-Gallardo S., Mönckeberg M., Ong J., Choolani M., Illanes S.E., Nardocci G. (2021). The Role of Long Non-Coding RNAs in Trophoblast Regulation in Preeclampsia and Intrauterine Growth Restriction. Genes.

[123] Kannampuzha S., Ravichandran M., Mukherjee A.G., Wanjari U.R., Renu K., Vellingiri B., Iyer M., Dey A., George A., Gopalakrishnan A.V. (2022). The mechanism of action of non-coding RNAs in placental disorders. Biomed. Pharmacother..

[124] Cech T.R., Steitz J.A. (2014). The Noncoding RNA Revolution—Trashing Old Rules to Forge New Ones. Cell.

[125] Lekchnov E.A., Zaporozhchenko I.A., Morozkin E.S., Bryzgunova O.E., Vlassov V.V., Laktionov P.P. (2016). Protocol for miRNA isolation from biofluids. Anal. Biochem..

[126] Kosaka N., Iguchi H., Yoshioka Y., Takeshita F., Matsuki Y., Ochiya T. (2010). Secretory mechanisms and intercellular transfer of MicroRNAs in living cells. J. Biol. Chem..

[127] Chen X., Ba Y., Ma L., Cai X., Yin Y., Wang K., Guo J., Zhang Y., Chen J., Guo X. (2008). Characterization of microRNAs in serum: A novel class of biomarkers for diagnosis of cancer and other diseases. Cell Res..

[128] Fu G., Brkić J., Hayder H., Peng C. (2013). MicroRNAs in Human Placental Development and Pregnancy Complications. Int. J. Mol. Sci..

[129] Sun N., Qin S., Zhang L., Liu S. (2021). Roles of noncoding RNAs in preeclampsia. Reprod. Biol. Endocrinol..

[130] Xu P., Ma Y., Wu H., Wang Y.-L. (2021). Placenta-Derived MicroRNAs in the Pathophysiology of Human Pregnancy. Front. Cell Dev. Biol..

[131] Subramanian A., Weiss D., Nyhan K., Dewan A., Jukic A.M.Z. (2022). Circulating miRNAs in the first trimester and pregnancy complications: A systematic review. Epigenetics.

[132] Li Q., Han Y., Xu P., Yin L., Si Y., Zhang C., Meng Y., Feng W., Pan Z., Gao Z. (2020). Elevated microRNA-125b inhibits cytotrophoblast invasion and impairs endothelial cell function in preeclampsia. Cell Death Discov..

[133] Licini C., Avellini C., Picchiassi E., Mensà E., Fantone S., Ramini D., Tersigni C., Tossetta G., Castellucci C., Tarquini F. (2020). Pre-eclampsia predictive ability of maternal miR-125b: A clinical and experimental study. Transl. Res..

[134] Tang J., Wang D., Lu J., Zhou X. (2021). MiR-125b participates in the occurrence of preeclampsia by regulating the migration and invasion of extravillous trophoblastic cells through STAT3 signaling pathway. J. Recept. Signal Transduct..

[135] Hromadnikova I., Kotlabova K., Ivankova K., Krofta L. (2017). First trimester screening of circulating C19MC microRNAs and the evaluation of their potential to predict the onset of preeclampsia and IUGR. PLoS ONE.

[136] Hromadnikova I., Kotlabova K., Hympanova L., Doucha J., Krofta L. (2014). First trimester screening of circulating C19MC mi-croRNAs can predict subsequent onset of gestational hypertension. PLoS ONE.

[137] Ura B., Feriotto G., Monasta L., Bilel S., Zweyer M., Celeghini C. (2014). Potential role of circulating microRNAs as early markers of preeclampsia. Taiwan. J. Obstet. Gynecol..

[138] Martinez-Fierro M.L., Garza-Veloz I. (2021). Analysis of Circulating microRNA Signatures and Preeclampsia Development. Cells.

[139] Martinez-Fierro M.L., Carrillo-Arriaga J.G., Luevano M., Lugo-Trampe A., Delgado-Enciso I., Rodriguez-Sanchez I.P., Garza-Veloz I. (2019). Serum levels of miR-628-3p and miR-628-5p during the early pregnancy are increased in women who subsequently develop preeclampsia. Pregnancy Hypertens..

[140] Miura K., Higashijima A., Murakami Y., Tsukamoto O., Hasegawa Y., Abe S., Fuchi N., Miura S., Kaneuchi M., Masuzaki H. (2015). Circulating chromosome 19 miRNA cluster microRNAs in pregnant women with severe pre-eclampsia. J. Obstet. Gynaecol. Res..

[141] Kim S.H., MacIntyre D.A., Binkhamis R., Cook J., Sykes L., Bennett P.R., Terzidou V. (2020). Maternal plasma miRNAs as potential biomarkers for detecting risk of small-for-gestational-age births. EBioMedicine.

[142] Cook J., Bennett P.R., Kim S.H., Teoh T.G., Sykes L., Kindinger L.M., Garrett A., Binkhamis R., MacIntyre D.A., Terzidou V. (2019). First Trimester Circulating MicroRNA Biomarkers Predictive of Subsequent Preterm Delivery and Cervical Shortening. Sci. Rep..

[143] Juchnicka I., Kuźmicki M., Niemira M., Bielska A., Sidorkiewicz I., Zbucka-Krętowska M., Krętowski A.J., Szamatowicz J. (2022). miRNAs as Predictive Factors in Early Diagnosis of Gestational Diabetes Mellitus. Front. Endocrinol..

[144] Geng Y., Ju Y., Ren F., Qiu Y., Tomita Y., Tomoeda M., Chang Z. (2014). Insulin receptor substrate 1/2 (IRS1/2) regulates Wnt/β-Catenin sig-naling through blocking autophagic degradation of dishevelled. J. Biol. Chem..

[145] Hubal M.J., Nadler E.P., Ferrante S.C., Barberio M.D., Suh J., Wang J., Dohm G.L., Pories W.J., Mietus-Snyder M., Freishtat R.J. (2016). Circulating adipocyte-derived exosomal MicroRNAs associated with decreased insulin resistance after gastric bypass. Obesity.

[146] Zhu Y., Tian F., Li H., Zhou Y., Lu J., Ge Q. (2015). Profiling maternal plasma microRNA expression in early pregnancy to predict gestational diabetes mellitus. Int. J. Gynecol. Obstet..

[147] Cao Y.L., Jia Y.J., Xing B.H., Shi D.D., Dong X.J. (2017). Plasma microRNA-16-5p, -17-5p and -20a-5p: Novel diagnostic biomarkers for gestational diabetes mellitus. J. Obstet. Gynaecol. Res..

[148] Kitamura T. (2013). The role of FOXO1 in β-cell failure and type 2 diabetes mellitus. Nat. Rev. Endocrinol..

[149] Collares C.V., Evangelista A.F., Xavier D.J., Rassi D.M., Arns T., Foss-Freitas M.C., Donadi E.A. (2013). Identifying common and specific mi-croRNAs expressed in peripheral blood mononuclear cell of type 1, type 2, and gestational diabetes mellitus patients. BMC Res Notes..

[150] Liang Y.Z., Dong J., Zhang J., Wang S., He Y., Yan Y.X. (2018). Identification of neuroendocrine stress response-related circulating Mi-croRNAs as biomarkers for type 2 diabetes mellitus and insulin resistance. Front. Endocrinol..

[151] Wilusz J.E., Sunwoo H., Spector D.L. (2009). Long noncoding RNAs: Functional surprises from the RNA world. Genes Dev..

[152] Marinescu M.C., Lazar A.L., Marta M.M., Cozma A., Catana C.S. (2022). Non-Coding RNAs: Prevention, Diagnosis, and Treatment in Myocardial Ischemia–Reperfusion Injury. Int. J. Mol. Sci..

[153] Beylerli O., Gareev I., Sufianov A., Ilyasova T., Guang Y. (2022). Long noncoding RNAs as promising biomarkers in cancer. Non-Coding RNA Res..

[154] Moradi M.-T., Rahimi Z., Vaisi-Raygani A. (2019). New insight into the role of long non-coding RNAs in the pathogenesis of preeclampsia. Hypertens. Pregnancy.

[155] Peñailillo R., Monteiro L.J., Acuña-Gallardo S., García F., Velásquez V., Correa P., Díaz P., Valdebenito P.P., Navarro C., Romero R. (2022). Identification of LOC101927355 as a Novel Biomarker for Preeclampsia. Biomedicines.

[156] Filardi T., Catanzaro G., Mardente S., Zicari A., Santangelo C., Lenzi A., Morano S., Ferretti E. (2020). Non-Coding RNA: Role in Gestational Diabetes Pathophysiology and Complications. Int. J. Mol. Sci..

[157] Jiang W., Sun X., Liu F., Cheng G., Li S., Xu M., Wu Y., Wang L. (2023). Circulating lncRNAs NONHSAT054669.2 and ENST00000525337 can be used as early biomarkers of gestational diabetes mellitus. Exp. Biol. Med..

[158] Su R., Wu X., Ke F. (2021). Long Non-Coding RNA HOTAIR Expression and Clinical Significance in Patients with Gestational Diabe-tes. Int. J. Gen. Med..

[159] Zhang Y., Wu H., Wang F., Ye M., Zhu H., Bu S. (2018). Long non-coding RNA MALAT1 expression in patients with gestational diabetes mellitus. Int. J. Gynecol. Obstet..

[160] Fickling S., Williams D., Vallance P., Nussey S., Whitley G. (1993). Plasma concentrations of endogenous inhibitor of nitric oxide synthesis in normal pregnancy and pre-eclampsia. Lancet.

[161] Maeda T., Yoshimura T., Okamura H. (2003). Asymmetric Dimethylarginine, an Endogenous Inhibitor of Nitric Oxide Synthase, in Maternal and Fetal Circulation. J. Soc. Gynecol. Investig..

[162] Williams D.J., Vallance P.J., Neild G.H., Spencer J.A., Imms F.J., Charkoudian N., Usselman C.W., Skow R.J., Staab J.S., Julian C.G. (1997). Nitric oxide-mediated vasodilation in human pregnancy. Am. J. Physiol. Circ. Physiol..

[163] A Khalil A., Tsikas D., Akolekar R., Jordan J., Nicolaides K.H. (2011). Asymmetric dimethylarginine, arginine and homoarginine at 11–13 weeks’ gestation and preeclampsia: A case–control study. J. Hum. Hypertens..

[164] Bian Z., Shixia C., Duan T. (2015). First-Trimester Maternal Serum Levels of sFLT1, PGF and ADMA Predict Preeclampsia. PLoS ONE.

[165] Cardaropoli S., Paulesu L., Romagnoli R., Ietta F., Marzioni D., Castellucci M., Rolfo A., Vasario E., Piccoli E., Todros T. (2012). Macrophage Migration Inhibitory Factor in Fetoplacental Tissues from Preeclamptic Pregnancies with or without Fetal Growth Restriction. J. Immunol. Res..

[166] Marzioni D., Fiore G., Giordano A., Nabissi M., Florio P., Verdenelli F., Petraglia F., Castellucci M. (2005). Placental Expression of Substance P and Vasoactive Intestinal Peptide: Evidence for a Local Effect on Hormone Release. J. Clin. Endocrinol. Metab..

[167] Marzioni D., Crescimanno C., Zaccheo D., Coppari R., Underhill C., Castellucci M. (2009). Hyaluronate and CD44 expression patterns in the human placenta throughout pregnancy. Eur. J. Histochem..

[168] Tossetta G., Fantone S., Giannubilo S.R., Busilacchi E.M., Ciavattini A., Castellucci M., Di Simone N., Mattioli-Belmonte M., Marzioni D. (2018). Pre-eclampsia onset and SPARC: A possible involvement in placenta development. J. Cell. Physiol..

[169] Phoswa W.N., Khaliq O.P. (2021). The Role of Oxidative Stress in Hypertensive Disorders of Pregnancy (Preeclampsia, Gestational Hypertension) and Metabolic Disorder of Pregnancy (Gestational Diabetes Mellitus). Oxidative Med. Cell. Longev..

[170] Weissgerber T.L., Mudd L.M. (2015). Preeclampsia and Diabetes. Curr. Diabetes Rep..

[171] Artemieva K.A., Stepanova Y.V., Stepanova I.I., Shamarakova M.V., Tikhonova N.B., Nizyaeva N.V., Tsakhilova S.G., Mikhaleva L.M. (2023). Morfofunctional and Molecular Changes in Placenta and Peripheral Blood in Preeclampsia and Gestational Diabetes Mellitus. Dokl. Biol. Sci..

[172] Rattila S., Kleefeldt F., Ballesteros A., Beltrame J.S., Ribeiro M.L., Ergün S., Dveksler G. (2020). Pro-angiogenic effects of pregnancy-specific glycoproteins in endothelial and extravillous trophoblast cells. Reproduction.

